# Supplementary material for: A model of COVID-19 pandemic with vaccines and mutant viruses
Source: PLoS One. 2022 Oct 24;17(10):e0275851. doi: 10.1371/journal.pone.0275851 (PMC9591069; doi:10.1371/journal.pone.0275851)
Supplement: S1 Text — (PDF) [file pone.0275851.s002.pdf]

An explanation to the construction of the model.

In the paper, we defined spontaneous and nonspontaneous transitions in Figure 1. In addition, Table 1 describe the types of transition of each node and the transition rates. Using this, we can derive the following system of differential equations:

$$\frac{d}{dt}S(t) = \Lambda - \beta\frac{S(t)I(t)}{N} - \beta^m\frac{S(t)I_m(t)}{N} - \alpha_1(t)S(t) - \nu S(t) \tag{1}$$

$$\frac{d}{dt}E(t) = \beta\frac{S(t)I(t)}{N} + \delta_1\beta\frac{V_1(t)I(t)}{N} + \delta_2\beta\frac{V_2(t)I(t)}{N} + \delta_3\beta\frac{V_3(t)I(t)}{N} - \sigma E(t) - \nu E(t) \tag{2}$$

$$\frac{d}{dt}E_m(t) = \beta^m\frac{S(t)I_m(t)}{N} + \delta_1^m\beta^m\frac{V_1(t)I_m(t)}{N} + \delta_2^m\beta^m\frac{V_2(t)I_m(t)}{N} + \delta_3^m\beta^m\frac{V_3(t)I_m(t)}{N} \tag{3}$$

$$- \sigma E_m(t) - \nu E_m(t) \tag{4}$$

$$\frac{d}{dt}I(t) = \sigma E(t) - (\eta_1 + \eta_2 + \eta_3)\mu I(t) - \nu I(t) \tag{5}$$

$$\frac{d}{dt}I_m(t) = \sigma E_m(t) - (\eta_1^m + \eta_2^m + \eta_3^m)\mu I_m(t) - \nu I_m(t) \tag{6}$$

$$\frac{d}{dt}H_k(t) = \eta_k\mu I(t) + \eta_k^m\mu I_m(t) - \gamma_k H_k(t) - \nu H_k(t) \tag{7}$$

$$\frac{d}{dt}R_k(t) = \gamma_k H_k(t) - \nu R_k(t) \tag{8}$$

$$\frac{d}{dt}V_1(t) = \alpha_1(t)S(t) - \alpha_2(t)V_1(t) - \delta_1\beta\frac{V_1(t)I(t)}{N} - \delta_1^m\beta^m\frac{V_1(t)I_m(t)}{N} - \nu V_1(t) \tag{9}$$

$$\frac{d}{dt}V_2(t) = \alpha_2(t)V_1(t) - \alpha_3(t)V_2(t) - \delta_2\beta\frac{V_2(t)I(t)}{N} - \delta_2^m\beta^m\frac{V_2(t)I_m(t)}{N} - \nu V_2(t) \tag{10}$$

$$\frac{d}{dt}V_3(t) = \alpha_3(t)V_2(t) - \delta_3\beta\frac{V_3(t)I(t)}{N} - \delta_3^m\beta^m\frac{V_3(t)I_m(t)}{N} - \nu V_3(t), \tag{11}$$

where $k = 1, 2, 3$, $\eta_1 + \eta_2 + \eta_3 = 1$, and $\beta^m = \tau\beta$. We have attached Table1 for explanation. First, Equation (1) represent the transition of individuals belonging to the susceptible compartment. Newly born individuals (Λ) are added to the susceptible compartment. In addition, susceptible individuals are converted at a rate of β (β^m) upon contact with individuals infected with the original virus (mutant virus), respectively. According to model assumption #4 in the paper, susceptible individuals who come into contact with an individual with the original virus (mutant virus) are moved to the E (E_m) compartment, respectively. Table 1 shows that this transition is a nonspontaneous transition. In addition, susceptible individuals can receive the primary vaccine or die (a natual death) at the ratio of $\alpha_1(t)$ and ν, respectively. $\frac{d}{dt}S(t)$ is the dynamical amount of individuals that are susceptible individuals over time. Therefore, we obtain the following equation.

$$\frac{d}{dt}S(t) = \Lambda - \beta\frac{S(t)I(t)}{N} - \beta^m\frac{S(t)I_m(t)}{N} - \alpha_1(t)S(t) - \nu S(t).$$

Next is the change over time of the exposed individuals. Individuals entering the exposed compartment are susceptible individuals and vaccinated individuals, as shown in the Table 1. In particular, individuals who were vaccinated in our model are divided into groups that received the primary, the secondary, and the booster shot vaccination. In Equation (1), individuals exposed to compartment I in the susceptible group enter compartment E. Similarly, vaccinated individuals come into contact with the I compartment and enter the E compartment at the rate specified in Table 1. Here, the transmission rate of $V_i \to E$ is $\delta_i \times \beta$ for $i = 1, 2, 3$. (δ_i is the probability that the i-th vaccination does not form antibodies.) These transmission rates reflect model assumption #2 in the paper. (We used this assumption to indicate a breakthrough infection.) That is, individuals vaccinated in contact with the I group are converted to the E compartment when no antibodies are formed. Conversely,

individuals in the E group convert spontaneously to the I group at a rate of μ over time. If we take into account those who die naturally, we obtain the Equation (2).

$$\frac{d}{dt}E(t) = \beta\frac{S(t)I(t)}{N} + \delta_1\beta\frac{V_1(t)I(t)}{N} + \delta_2\beta\frac{V_2(t)I(t)}{N} + \delta_3\beta\frac{V_3(t)I(t)}{N} - \sigma E(t) - \nu E(t).$$

The change over time of E_m can be obtained similar to that of E. However, it is always $\delta_i^m < \delta_i$ by model assumption #6. Equations (5) - (8) are all spontaneous transitions and can be easily obtained by referring to the Table 1 considering natural death.

Changes over time in V_i are typically divided into individuals who receive the i-th vaccination and individuals who change into exposed groups (E and E_m) for $i = 1, 2, 3$. Individuals changing to exposed group E are expressed as decreasing by $\delta_i\beta\frac{V_i(t)I(t)}{N}$, as opposed to that described in Equation (2). Thus, individuals entering V_1 come from S, individuals entering V_2 come from V_1, and individuals entering V_3 come from V_2. Specifically, V_1 and V_2 take into account both outgoing and incoming individuals following vaccination. For convenience of notation, if S is denoted as V_0, we can obtain the following equation for $i = 1, 2$:

$$\frac{d}{dt}V_i(t) = \alpha_i(t)V_{i-1}(t) - \alpha_{i+1}(t)V_i(t) - \delta_i\beta\frac{V_i(t)I(t)}{N} - \delta_1^m\beta^m\frac{V_i(t)I_m(t)}{N} - \nu V_i(t).$$

Since there are no individuals transitioning to the next vaccination compartment at $i = 3$, the change over time of the V_3 compartment is as follows:

$$\frac{d}{dt}V_3(t) = \alpha_3(t)V_2(t) - \delta_3\beta\frac{V_3(t)I(t)}{N} - \delta_3^m\beta^m\frac{V_3(t)I_m(t)}{N} - \nu V_3(t).$$

Table 1: **Entering and exiting transitions of each node.**

**Node**	**Entering**	**Entering Rate**	**Transition's type**	**Exiting**	**Exiting rate**	**Transition's type**
S	-	-	-	$S \to V_1$	$\alpha_1(t)$	Spontaneous
				$S \to E$	β	Nonspontaneous
				$S \to E_m$	β^m	Nonspontaneous
E	$S \to E$	β	Nonspontaneous	$E \to I$	σ	Spontaneous
	$V_1 \to E$	$\delta_1 \times \beta$	Nonspontaneous			
	$V_2 \to E$	$\delta_2 \times \beta$	Nonspontaneous			
	$V_3 \to E$	$\delta_3 \times \beta$	Nonspontaneous			
E_m	$S \to E_m$	β^m	Nonspontaneous	$E_m \to I_m$	σ	Spontaneous
	$V_1 \to E_m$	$\delta_1^m \times \beta^m$	Nonspontaneous			
	$V_2 \to E_m$	$\delta_2^m \times \beta^m$	Nonspontaneous			
	$V_3 \to E_m$	$\delta_3^m \times \beta^m$	Nonspontaneous			
I	$E \to I$	σ	Spontaneous	$I \to H_k$	$\eta_k \times \mu$	Spontaneous
I_m	$E_m \to I_m$	σ	Spontaneous	$I_m \to H_k$	$\eta_k^m \times \mu$	Spontaneous
H_k	$I \to H_k$	$\eta_k \times \mu$	Spontaneous	$H_k \to R_m$	γ_k	Spontaneous
	$I_m \to H_k$	$\eta_k^m \times \mu$	Spontaneous			
R_k	$H_k \to R_k$	γ_k	Spontaneous	-	-	-
V_1	$S \to V_1$	$\alpha_1(t)$	Spontaneous	$V_1 \to V_2$	$\alpha_2(t)$	Spontaneous
				$V_1 \to E$	$\delta_1 \times \beta$	Nonspontaneous
				$V_1 \to E_m$	$\delta_1^m \times \beta^m$	Nonspontaneous
V_2	$V_1 \to V_2$	$\alpha_2(t)$	Spontaneous	$V_2 \to V_3$	$\alpha_3(t)$	Spontaneous
				$V_2 \to E$	$\delta_2 \times \beta$	Nonspontaneous
				$V_2 \to E_m$	$\delta_2^m \times \beta^m$	Nonspontaneous
V_3	$V_2 \to V_3$	$\alpha_3(t)$	Spontaneous	$V_3 \to E$	$\delta_3 \times \beta$	Nonspontaneous
				$V_3 \to E_m$	$\delta_3^m \times \beta^m$	Nonspontaneous

Table 1 shows the transitions entering and exiting each node and each transition's type.

Model assumptions

1. Because the COVID-19 pandemic has manifested for a long period, our model considers the natural mortality and birth rates of the population in the course of the pandemic.

2. An individual who has been vaccinated but has not developed antibodies can be infected. When a vaccine does not provide complete immunity to the virus and the vaccinated individual becomes infected with the disease, it is called “breakthrough infection” [34].

3. Individuals infected with one strain develop immunity against other mutations of the same strain. That is, we rule out reinfection, which means an individual who has recovered from the original virus becomes immune to the mutant virus and vice versa. This assumption was adopted in [27]. Reinfection with the same strain is also excluded.

4. Infection by a mutant virus occurs through contact with an individual infected with a mutant virus and infection by the original virus occurs through contact with an individual infected with the original virus. More specifically, a susceptible individual who comes into contact with an individual infected with the original virus may or may not become infected with the original virus, but they do not become an individual infected with the mutant virus.

5. Hospitalized individuals include those who are hospitalized as well as those who are self-isolating. Individuals in this group do not develop secondary infections.

6. The vaccine’s efficacy against the mutant is slightly inferior than that against the original virus [35-38].
