# Supplementary material for: A model of COVID-19 pandemic with vaccines and mutant viruses
Source: PLoS One. 2022 Oct 24;17(10):e0275851. doi: 10.1371/journal.pone.0275851 (PMC9591069; doi:10.1371/journal.pone.0275851)
Supplement: S2 Text — (PDF) [file pone.0275851.s003.pdf]

Proof of Proposition 1.

Proposition 1. *If* D^0 *is the* DFE *state of the SEIHR model (2), then* D^0 *is locally asymptotically stable if* $R_0 = \rho(G) \leq 1$*, but unstable if* $R_0 > 1$*.*

Proof. The proof of this proposition proceeds similarly to the derivation of the basic reproduction number of the *SEIR* model of the study [44]. Since $X = (S, E, I, H_1, H_2, H_3, R_1, R_2, R_3) = D^0$, we can assume that the susceptible group S is almost similar to the total population N, i.e., $S \approx N$. Therefore we can obtain the linear equations for $E(t)$ and $I(t)$:

$$\begin{aligned} E'(t) &= \beta I(t) - \sigma E(t) \\ I'(t) &= \sigma E(t) - \mu I(t). \end{aligned} \tag{1}$$

In order to easily handle the derivative term, we use the Laplace transforms of $E(t)$ and $I(t)$:

$$\begin{aligned} \widetilde{E}(\lambda) &= \int_0^\infty E(t) e^{-\lambda t} dt \\ \widetilde{I}(\lambda) &= \int_0^\infty I(t) e^{-\lambda t} dt \end{aligned} \tag{2}$$

with $\mathrm{Re}(\lambda) > 0$. Now, we apply the Laplace transforms on both sides of (1), then

$$\begin{aligned} \mathcal{L}(E'(t)) &= \int_0^\infty E'(t) e^{-\lambda t} dt \\ &= \left[E(t) e^{-\lambda t}\right]_{t=0}^{t=\infty} + \lambda \int_0^\infty E(t) e^{-\lambda t} dt \\ &= -E(0) + \lambda \widetilde{E}(\lambda) = \beta \widetilde{I}(\lambda) - \sigma \widetilde{E}(\lambda), \end{aligned} \tag{3}$$

and similarly, we can calculate the Laplace transform of $I'(t)$:

$$\mathcal{L}(I'(t)) = -I(0) + \lambda \widetilde{I}(\lambda) = \sigma \widetilde{E}(\lambda) - \mu \widetilde{I}(\lambda). \tag{4}$$

where $E(0)$ and $I(0)$ are the initial conditions for $E(t)$ and $I(t)$, respectively. By the equations (3) and (4), we obtain a linear system,

$$\begin{pmatrix} -\sigma - \lambda & \beta \\ \sigma & -\mu - \lambda \end{pmatrix} \begin{pmatrix} \widetilde{E}(\lambda) \\ \widetilde{I}(\lambda) \end{pmatrix} = \begin{pmatrix} E(0) \\ I(0) \end{pmatrix}. \tag{5}$$

The eigenvalues λ are the determinant of the matrix,

$$\begin{vmatrix} -\sigma - \lambda & \beta \\ \sigma & -\mu - \lambda \end{vmatrix} = 0. \tag{6}$$

It implies that

$$\begin{aligned} & \lambda^2 + (\sigma + \mu)\lambda + \sigma(\mu - \beta) = 0 \\ \Longrightarrow \quad & \lambda_\pm = \frac{-(\sigma + \mu)}{2} \pm \frac{\sqrt{(\sigma - \mu)^2 + 4\sigma\beta}}{2}. \end{aligned} \tag{7}$$

Since σ, $\beta > 0$,then $(\sigma - \mu)^2 + 4\sigma\beta$ has always positive sign, that is, the eigenvalues are real.

1. If $R_0 = \beta/\mu < 1$, then both $\lambda_+ < 0$ and $\lambda_- < 0$. Therefore the solution decays exponentially.
2. If $R_0 = \beta/\mu > 1$, then $\lambda_+ > 0$ and $\lambda_- < 0$. So the solution grows exponentially.
3. If $R_0 = \beta/\mu = 1$, then $\lambda_+ = 0$ and $\lambda_- = -(\sigma + \mu)$. The solution remains constant.

□
