# Supplementary material for: A model of COVID-19 pandemic with vaccines and mutant viruses
Source: PLoS One. 2022 Oct 24;17(10):e0275851. doi: 10.1371/journal.pone.0275851 (PMC9591069; doi:10.1371/journal.pone.0275851)
Supplement: Attachment — Submitted filename: Plos_One_Review.pdf [file pone.0275851.s005.pdf]

Reviewer's Comment

Dr. Shyam Sumanta Das

July 19, 2022

Article: PONE-D-22-14438
A model of COVID-19 pandemic with vaccines and mutant viruses

The author has worked on a model of the COVID-19 pandemic with the presence of multiple mutant viruses and studied the effect of vaccination on diease propagation in South Korea. They have developed a mathematical model based on the SEIR type of compartment model in which they have added the various compartments related to multiple vaccinations and mutant virus of COVID-19. The author has calculated the effective reproduction numbers and equilibrium points of the original virus, Delta, and Omricon strains of the SARS-CoV-2 virus. Further, the author has carried out numerical simulations for the different cases and fit the model with the observed data from the confirmed cases from South Korea. The author has did a good work. I will suggest the following modifications in the manuscript.

1. I will suggest the author to rewrite the introduction . In the introduction , the author should cite some more literatures related to modeling of COVID-19 disease transmission with the presence of multiple mutant viruses and the effect of vaccination. Also, the author should include some works related to multistrain dynamics of various strains of SARS-CoV-2 virus.

2. In the method part, it is not necessary to explain about SIR and SEIR model. The author can directly start explaining the proposed SEIHRV model which is based on SEIR framework. They can cite the original reference of SEIR model while writing the method.

3. The author should explain little bit about the breakthrough infection in the model.

4. In Figure 1., the author should slightly modify the schematic diagram for SEIHRV model. There is an extra I_m compartment near the vaccine compartments (V_1,V_2 and V_3).

5. As the COVID-19 pandemic is endemic now in the world, I think the author should include the demographic information in model such as birth rate and death rate.

6. In Figure. 11, the author should mentioned the values of τ each of the sub-figures from 1.1 to 1.8.
