# Supplementary material for: A model of COVID-19 pandemic with vaccines and mutant viruses
Source: PLoS One. 2022 Oct 24;17(10):e0275851. doi: 10.1371/journal.pone.0275851 (PMC9591069; doi:10.1371/journal.pone.0275851)
Supplement: Attachment — Submitted filename: Response to Reviewers.pdf [file pone.0275851.s006.pdf]

Response to Reviewers

We are very grateful to anonymous referees for the extensive and insightful comments regarding our paper, “A model of COVID-19 pandemic with vaccines and mutant viruses”. We agreed with most of the referees’ comments on the earlier version of this paper. We believe that the paper has greatly improved as a result of incorporating them. Responses to specific comments are itemized below.

1 Reviewer 1

The authors proposed an extended SIR with mutation and multi-vaccine model. They claim to fit the model for the case of Korea for COVID-19.

In summary, the idea is good and implementation is well done. However, the text should be improved. I hope my comments would help the authors to improve their work.

Major:

1. **The text is hard to read due to the grametical and lingustic issues currently found in the paper. The authors suggested to use a native English speaker to fix these issues in the text.**

Answer We have had a native-speaking expert to review the manuscript and edit it accordingly. Now, the text seems easier to read.

2. **The review pargaraph in the Introduction section is limited. The authors need to disucss recent works about multi-strain and multi-mutation models and more models that combine SIR and vaccination.**

   In particualr, this journal and others have several recent such publications:

   A. Marquioni and de Aguiar, Modeling neutral viral mutations in the spread of SARS-CoV-2 epidemics (2021) - Plos one.

   B. Lazebnik and Bunimovich-Mendrazitsky, Generic Approach For Mathematical Model of Multi-Strain Pandemics, (2022) - Plos one.

   C. Gordo et al., Genetic Diversity in the SIR Model of Pathogen Evolution (2009) - Plos One.

   D. Arruda et al., Modelling and optimal control of multi strain epidemics, with application to COVID-19 (2021) - Plos one.

   E. Lazebnik and Blumrosen, Advanced Multi-Mutation with Intervention Policies Pandemic Model (2022) - IEEE Acsses.

   F. Khyar and Allali, Global dynamics of a multi-strain SEIR epidemic model with general incidence rates: application to COVID-19 pandemic (2020) - Nonlinear Dynamics.

Answer The studies suggested by the reviewers are of particular relevance to our study. We included the above papers and other recent ones in Introduction.

→ Please refer to Lines 29 - 51.

“Two types of extensions are of particular relevance to our study. ... Lazebnik et al. provided an advanced multi-mutation model that considers intervention policies including vaccination and lockdown [31].”

3. **The novelty of the work is not clear from the Abstract or the Introduction sections.**

Answer Rewrote the Abstract and Introduction sections as follows:

- **In Abstract**: In particular, our model consider breakthrough infection according to the antibody formation rate following multiple vaccinations. We obtain the effective reproduction numbers of the original virus, the Delta, and the Omicron variants by fitting this model to data in Korea. Additionally, we provide various simulations adjusting the daily vaccination rate and the timing of vaccination to investigate the effects of these two vaccine-related measures on the number of infected individuals. In the sensitivity analysis on the vaccination rate of Korean data, it is shown that a 10% increase (decrease) in vaccination rates can reduce the number of confirmed cases by 35.22% (increases by 82.82%), respectively.
- **In Introduction (Lines 52-70)**: We developed a mathematical model based on the *SEIR* type of compartment model into which we added various compartments related to multiple vaccinations and mutant viruses of COVID-19. The difference from other studies is that the multiple vaccination compartments were designed to consider the antibody formation rates of the original virus and the mutant virus according to the number of vaccinations. Additionally, to consider breakthrough infections, we assumed that vaccinated individuals can also become infected with the original or mutated strain if they do not develop antibodies even after being vaccinated. We calculated the effective reproduction numbers of the original virus and the Delta variant. Through this, we found the critical level of effective reproduction number over which the mutant virus becomes prevalent using Korean data. The strength of our model is that we control the proportion of susceptible individuals who transfer to vaccination compartments daily, allowing simulations with the number of daily vaccinations and initial inoculation date. As a specific example, we showed the efficacy of the vaccine by providing a sensitivity analysis that changed the vaccination rate through model fitting with actual data obtained between March 12, 2020 and December 31, 2021 in Korea. We hope that the results of these simulations help make rational decision-making by those who are reluctant to vaccination.

4. **"It assigns a population to labeled compartments – for example, S, I, or R, (Susceptible, Infectious, or Recovered)." - bad wording and cite! this is not your idea...**

Answer We deleted the above phrase and cited the SIR-related studies in the Introduction section of our manuscript.

→ "The susceptible-infected-removed (SIR) model focuses on disease compartments that was developed in 1927 by Kermack and McKendrick and has been used as a basic mathematical model [20]. The SIR model has been expanded to consider an incubation period, reinfection, and quarantining, see [21–24] for more details." (Lines 24-28)

5. **I do not see any point in giving so much attention to Eqs. 1 + 2, you can remove this sub-section...**

Answer As advised, we removed this subsection. Descriptions on the studies related to SIR and SEIR have been moved to the Introduction section.

6. **An explanation to the construction of Eq. (3) should be provided (even in the appendix).**

Answer This information has been added to Supporting information.

7. **Proposition 1 - we saw similar results in multiple works in the past - seems redundant to me. Cite**

Answer The proof Proposition 1 proceed in a similar way to that in Piovella, N. (2020). We cited this paper and moved the proof to Supporting information.

[44] Piovella, N. Analytical solution of SEIR model describing the free spread of the COVID-19 pandemic. Chaos, Solitons & Fractals. 2020;140: 110243.

8. **Line 281 - why? you should explain these parameter values.**

Answer We used parameter values based on Korean data. These are summarized in Table 4 (Line 261) and references are cited.

9. **The results can be presented better. A lot of the graphs repeating themselves and do not provide any additional insights. The authors should present a "meta" analysis of these results to the reader.**

Answer We added more explanations about the graphs. We also tried to present a meta analysis of these results in the Discussion section.

10. **I think the "Cost and benefit analysis of vaccines" is out of the scope of the paper and the authors tried to introduce another "cool" result. I reccomend to delete it.**

Answer As advised, this part has been deleted and we rewrote it as a research limitation in the discussion section.

→ "An economic cost and benefit analysis of mitigating policies such as social distancing and vaccination can be conducted using the results of various simulations in this study. Specifically, we can examine whether the economic benefits of social distancing (or vaccination) exceed or fall short of its costs. Since the Korean government has changed the intensity of social distancing several times in the course of fighting the spread of COVID-19, it can be a natural experiment to estimate the benefits and costs of the various intensity of social distancing.." (Lines 624-630)

11. **The Discussion section just "reads" the results - it does not provide any insights to how policymakers can use this model and results.**

Answer We modified the Discussion section. We summarized the results obtained in the "Results" section and the facts that can lead to these results. The following is part of the Discussion section.

- We found in this experiment that non-pharmaceutical interventions lowered the number of confirmed cases and slowed the spread of transmission. A case where R_0 was actually reduced by the implemented policy was introduced in [50] and strategies for non-pharmaceutical interventions was introduced in [8]. (Lines 565-568)
- Even with vaccination, non-pharmaceutical interventions are still important. Figures 4, 6 show that with increasing R_0, we observe convergence to the unvaccinated state even with vaccination. As a detailed study related to this, there is a study [9]. (Lines 586-588)
- An interesting result of this experiment was that a new peak was produced when the effective reproduction number of the mutant virus was more than double that of the original virus. This could help us to allocate medical and administrative resources to two different viruses in the presence of mutant viruses. (Lines 591-594)
- As a result, we showed that a 10% change in vaccination rates can make a huge difference, reducing the number of confirmed cases by 35.22% or increasing it by 82.82%. These results may help inform people who are hesitant to get vaccinated. (Lines 609-612)

12. **The Discussion section lack an honest discussion of the research limitations.**

Answer We added the limitation paragraph of the study to the Discussion section. A major limitation of our study is that reinfection was not taken into account.

→ We acknowledge some limitations of the present study. Our model ruled out reinfection between the two strain because the rate of reinfection was low at the time of the Delta variant in Korea [53]. We conducted simulations for the Omicron variant, which was newly reported toward the end of November 2021, by changing the parameters of the model and confirmed that the Omicron variant has a very large effective reproduction number, as shown in Figure 15. However, reinfection rates of the Omicron variant have been reported to be high [53] and our model was not suitable to provide a fitting and prediction for this variant in the long term. Also, we did not consider antibodies that disappear over time.

This is very important for mutant viruses with high reinfection rates that may emerge in the future. We leave the model improvement considering reinfections for future research. (Lines 613-623)

13. **A fitting error analysis is missing.**

Answer We added a relative L^2 norm error to each fitting section.
→ Lines 444, 484, and 540

14. **I would like to see a "novelty statement" comparing the current work with previous multi-strain extended SIR models. I think the authors would see that the current "novelty statement" is somewhat problematic.**

Answer We added the novelty of our work in the Introduction section. Novelty of our study comparing the current work with previous multi-strain extended SIR models is as follow:

1) Many models for verifying the efficacy of a vaccine include a single vaccine compartment, or apply reduced disease transmission rate by a vaccine to the model. We added detailed vaccination compartments in the mathematical model.

2) Three additional vaccination compartments (the primary and the secondary vaccinations and booster shots) considered the antibody formation rate of the vaccine to reflect breakthrough infections.

3) The strength of our model is that we control the proportion of susceptible individuals who transfer to vaccination compartments daily, allowing simulations with the number of daily vaccinations and initial inoculation date. Our study includes various simulations related to this.

We have attached relevant parts of the manuscript in comment #3.

Minor:

1. **Intro, first statement - cite?**

Answer Severe acute respiratory syndrome coronavirus 2 (SARS-CoV-2), also known as COVID-19, emerged in late 2019 [1] and continues to threaten the world [2].

[1] Riou, J., Althaus, C. L. Pattern of early human-to-human transmission of Wuhan 2019 novel coronavirus (2019-nCoV), December 2019 to January 2020. Eurosurveillance. 2020;25(4): 2000058. doi: https://doi.org/10.2807/1560-7917.ES.2020.25.4.2000058

[2] Dong, E., Du, H., Gardner, L. An interactive web-based dashboard to track COVID-19 in real time. The Lancet infectious diseases 2020; 20(5), 533–534. doi: https://doi.org/10.1016/S1473-3099(20)30120-1

2. **Intro, second statement - cite?**

Answer The virus has reduced social activities such as traveling and interactions between individuals [3] and negatively impacted education [4] and the economy [5].

[3] UNWTO World Tourism Barometer and Statistical Annex, December 2020. UNWTO World Tourism Barometer (English version). 2020;18(7): 1–36. doi: 10.18111/wtobarometereng.2020.18.1.7

[4] UNESCO. Education: from school closure to recovery. Available: https://www.unesco.org/en/covid-19/education-response?hub=800

[5] World Bank. World Development Report 2022, Chapter 1. The economic impacts of the COVID-19 crisis. Available: https://www.worldbank.org/en/publication/wdr2022/brief/chapter-1-introduction-the-economic-impacts-of-the-covid-19-crisis

3. **Intro, "Using the above model, we provide various simulations" - no model is provided yet at this point.**

Answer We deleted that sentence.

4. **Intro, last paragraph is not clear to me. It seems you are all over the place and I was not able to follow your logical process.**

Answer We changed this paragraph.

5. **"There are various methods for mathematical modeling of infectious diseases." - cite?**

Answer We changed the sentence to the following and added a citation.

→Diverse mathematical models exist for infectious diseases [32].

[32] Diekmann, O., Heesterbeek, J. A. P. Mathematical epidemiology of infectious diseases: model building, analysis and interpretation (Vol. 5). John Wiley & Sons. Chichester; 2000

6. **"compartment model is one of the most representative mathematical modeling techniques" - cite?**

Answer We changed the sentence to the following and added a citation.

→ The compartment model is one of the representative mathematical modeling techniques [11].

[11] Banholzer, N., Van Weenen, E., Lison, A., Cenedese, A., Seeliger, A., Kratzwald, B., et al. Estimating the effects of non-pharmaceutical interventions on the number of new infections with COVID-19 during the first epidemic wave. PLoS one. 2021;16(6): e0252827. doi: https://doi.org/10.1371/journal.pone.0252827

7. **"To construct our model, we start with basic epidemic models which are the prototypical epidemic models." - what?**

Answer We deleted that sentence in revised version of manuscript.

8. **"As an epidemic occurs, the number of susceptible individuals decrease over time and eventually becomes zero." - not ture, please see https://doi.org/10.3390/sym13071120**

Answer We took a good look at the research you suggested for the reference. We deleted that sentence in revised version of manuscript and we cited that study in the introduction.

9. **"the number of secondary infections produced by one infected individual after the initial outbreak is called the effective reproduction number." - cite?**

Answer We cited the study [45].

[45] Zhao, S., Musa, S. S., Hebert, J. T., Cao, P., Ran, J., Meng, J., et al. Modelling the effective reproduction number of vector-borne diseases: the yellow fever outbreak in Luanda, Angola 2015–2016 as an example. PeerJ, 2020; 8: e8601. doi: https://doi.org/10.7717/peerj.8601

Positive:

1. Really likes tables 1 + 2.
2. The "An effective reproduction number" sub-section well presented.
3. The "Model fitting for Korea Coronavirus data and reproduction numbers" sub-section is fairly presented.

2 Reviewer 2

The authors propose an extension of the SEIR compartment model to describe the evolution of the COVID-19 epidemics. Many additional compartments are added to the model to include vaccinations (two doses plus booster shot), hospitalizations and mutant variants. After a careful analysis of main features of the model the authors fit the parameters to data from Korea, obtaining interesting results.

When I started reading the paper I thought it would be messy and hard to follow, given the large number of parameters needed to accommodate all compartments and the corresponding transition rates between them. However, the paper is really well written and organized. I like the analysis presented in the Results section and the way conclusions are drawn from the figures. I also enjoyed reading the section where the model parameters are fit to the Korean data and how the original+delta

strains are replaced by delta+omicron. I believe the model contributes significantly to advance the modeling of COVID-19 and deserves publication in PLoS One.

I have only a few minor suggestions that the authors should consider:

1. **item 3 of the "SEIHRV -mutant model" is confusing. I suggest simplifying it to The vaccine of the coronavirus (CoV) is effective for mutant viruses (MuV) and cross-reinfections are ruled out.**

Answer This comment was of great help to our manuscript. The original sentence said two things. Therefore, we describe each of these two facts separately. The sentences have been modified as follows:

- The vaccine's efficacy against the mutant is slightly inferior than that against the original virus [35–38].
- Individuals infected with one strain develop immunity against other mutations of the same strain. That is, we rule out reinfection, which means an individual who has recovered from the original virus becomes immune to the mutant virus and vice versa.

These sentences are cited in the studies below.

35 Sheikh A, McMenamin J, Taylor B, Robertson C. SARS-CoV-2 Delta VOC in Scotland: demographics, risk of hospital admission, and vaccine effectiveness. Lancet 2021; 397: 2461-2.

36 World Health Organization. Tracking SARS-CoV-2 variants [Internet]. Geneva: World Health Organization; 2021 [cited 2021 Jul 15].

37 Centers for Disease Control and prevention. Interim clinical considerations for use of COVID-19 vaccines currently authorized in the United States [Internet]. Atlanta: Centers for Disease Control and prevention; 2021 [cited 2021 Jul 15].

38 Ahn, Sang Hyun, and Seung Hwa Lee. "Updates on coronavirus disease 19 vaccine and its clinical application." Korean Journal of Family Practice 11.4 (2021): 236-246.

27 O. Khyar and K. Allali, "Global dynamics of a multi-strain SEIR epidemic model with general incidence rates: Application to COVID-19 pandemic," Nonlinear Dyn., vol. 102, no. 1, pp. 489509, Sep. 2020.

2. **the model assumes the all infected individuals are hospitalized. However, most people recover without need to go to a hospital. The authors could, therefore, mention that the lowest level of hospitalization could also be interpreted as "no-hospitalization" (and no cost).**

Answer During the period when the Delta variant was prevalent, almost everyone infected in Korea was hospitalized, even those with mild symptoms. Because at that time, the number of infections per day did not exceed 10,000. Also, some people who could not be hospitalized have self-quarantined. Therefore, our model did not take into account non-isolated people. To avoid confusion, it is noted that hospitalized individuals include individuals who are self-isolating. (Line 111, 176-177)

3. **I don't understand the need for Laplace transforms to prove proposition 1. The matrix in eq.(12) can be obtained directly from the coefficients of E and I in eq.(8), since the system is linear.**

Answer The proof you pointed out proceeded in a similar way to that in the study of Piovella, N. (2020). We cited this paper and moved the proof to Supporting information.

Piovella, N. (2020). Analytical solution of SEIR model describing the free spread of the COVID-19 pandemic. Chaos, Solitons & Fractals, 140, 110243.

4. **I find the notation VD for vaccination dose a bit confusing. Because VD is defined as the number of days it takes to vaccinate the entire population, the larger is VD the slower is the rate of vaccination. I understand this is convenient for the authors but it might be clearer to use 1/VD or maybe 1000/VD to have numbers of order 1. That would have a more direct interpretation, as large VD would mean large the**

rate of vaccination. But I leave it to the authors whether it is worth to change the notation.

Answer That's a good point. We've changed the VD to make it easier to understand to reflect your ideas. The manuscript has been modified as follows.

→ If we assume k is target period to allow the entire population to complete the first vaccination, then N/k is the number of daily vaccinations. For notational convenience, we denote the parameter $1/k$ as VD (Vaccination; Daily). (Line 292)

5. **line 370 - "Now we now"**

Answer That part has been revised.

6. **I don't think figure 14 is needed. Maybe it could me moved to the supplemental material.**

Answer We agree with you. The figure has been deleted.

7. **Figure 16 is very important. It is impressive how a change in 10% in the rate of vaccination could make such a large difference and probably save so many lives. This should be shown to all anti-vaccination groups.**

Answer Thank you for your evaluation. As you advised, we highlighted this result in the Abstract and Introduction sections.

3 Reviewer 3

The authors proposed an extended SIR with mutation and a multi-vaccine model. In general, the idea is good and the implementation is on top. I like their work. This paper has some publishing points, but there are several issues why this paper is not ready for publication.

Main:

1. **Extensive editing of English language and style required. The text is difficult to read due to grammatical and linguistic problems found in the article.**

Answer We have had a native-speaking expert to review the manuscript and edit it accordingly. Now, the text seems easier to read.

2. **The abstract does not report any findings, only an approach. What is the novelty of the manuscript?**

Answer Rewrote the Abstract and Introduction sections as follows:

- **In Abstract**: In particular, our model consider breakthrough infection according to the antibody formation rate following multiple vaccinations. We obtain the effective reproduction numbers of the original virus, the Delta, and the Omicron variants by fitting this model to data in Korea. Additionally, we provide various simulations adjusting the daily vaccination rate and the timing of vaccination to investigate the effects of these two vaccine-related measures on the number of infected individuals. In the sensitivity analysis on the vaccination rate of Korean data, it is shown that a 10% increase (decrease) in vaccination rates can reduce the number of confirmed cases by 35.22% (increases by 82.82%), respectively.
- **In Introduction (Lines 52-70)**: We developed a mathematical model based on the *SEIR* type of compartment model into which we added various compartments related to multiple vaccinations and mutant viruses of COVID-19. The difference from other studies is that the multiple vaccination compartments were designed to consider the antibody formation rates of the original virus and the mutant virus according to the number of vaccinations. Additionally, to consider breakthrough infections, we assumed that vaccinated individuals can also become infected with the original or mutated strain if they do not develop antibodies even after being vaccinated. We calculated the effective

reproduction numbers of the original virus and the Delta variant. Through this, we found the critical level of effective reproduction number over which the mutant virus becomes prevalent using Korean data. The strength of our model is that we control the proportion of susceptible individuals who transfer to vaccination compartments daily, allowing simulations with the number of daily vaccinations and initial inoculation date. As a specific example, we showed the efficacy of the vaccine by providing a sensitivity analysis that changed the vaccination rate through model fitting with actual data obtained between March 12, 2020 and December 31, 2021 in Korea. We hope that the results of these simulations help make rational decision-making by those who are reluctant to vaccination.

3. **The biological introduction and the review of existing modelling is insufficient. The authors should discuss recent work on multi-strain, multi-mutation models and other models combining SIR and vaccination.**

   There are several recent publications in PLOS one:

   D. Arruda et al., Modeling and optimal control of multi-strain epidemics with application to COVID-19 (2021) - Plos. one.

   A. Marchioni and de Aguiar, Modeling Neutral Viral Mutations in the Spread of SARS-CoV-2 Epidemics (2021) - Plos one.

   B. Lazebnik and Bunimovich-Mendrazitsky, A general approach to the mathematical model of multi-strain pandemics, (2022) - Plos one.

   C. Gordo et al., Genetic Diversity in the SIR Model of Pathogen Evolution (2009) - Plos One.

Answer he studies suggested by the reviewers are of particular relevance to our study. We included the above papers and other recent ones in Introduction.

→ Please refer to Lines 29 - 51.

"Two types of extensions are of particular relevance to our study. ... Lazebnik et al. provided an advanced multi-mutation model that considers intervention policies including vaccination and lockdown [31]."

4. **"It allocates the population to labeled compartments, such as S, I, or R, (susceptible, infectious, or recovered)." - bad wording and quote! it's not your idea...**

Answer We deleted the above phrase and cited the SIR-related studies in the Introduction section of our manuscript.

→ "The susceptible-infected-removed (SIR) model focuses on disease compartments that was developed in 1927 by Kermack and McKendrick and has been used as a basic mathematical model [20]. The SIR model has been expanded to consider an incubation period, reinfection, and quarantining, see [21–24] for more details." (Lines 24-28)

5. **The equations 1+2 are known, not need speak about it...**

Answer As advised, we removed this subsection. Descriptions on the studies related to SIR and SEIR have been moved to the Introduction section.

6. **Explanation of the equations (3) must be provided!.**

Answer This information has been added to Supporting information.

7. **N = 50, 000, 000, $\sigma = 1/4.1$ and $\mu = 1/4$ - you must explain these parameters!**

Answer We used parameter values based on Korean data. These are summarized in Table 4 (Line 261) and references are cited.

8. **No clear explanation of the graphs. Authors should provide analysis of these results.**

Answer We added more explanations about the graphs. Also, the notation can be confusing, so we changed the meaning of VD to the number of daily vaccinations. This was recommended by the other reviewer and it should have made the graph a little easier to understand. We have added some studies related to our results to the discussion section.

→ [50] Kucharski, A. J., Russell, T. W., Diamond, C., Liu, Y., Edmunds, J., Funk, S., et al. Early dynamics of transmission and control of COVID-19: a mathematical modelling study. The lancet infectious diseases. 2020; 20(5): 553–558. doi: https://doi.org/10.1016/S1473-3099(20)30144-4

[8] Ferguson, N. M., Laydon, D., Nedjati-Gilani, G., Imai, N., Ainslie, K., Baguelin, M., Ghani, A. C., et al. Impact of non-pharmaceutical interventions (NPIs) to reduce COVID-19 mortality and healthcare demand. 2020. doi: 10.25561/77482

[9] Gozzi, N., Bajardi, P., Perra, N. The importance of non-pharmaceutical interventions during the COVID-19 vaccine rollout. PLoS computational biology. 2021; 17(9): e1009346. doi: https://doi.org/10.1371/journal.pcbi.1009346

[52] Thunstr̈om, L., Newbold, S. C., Finnoff, D., Ashworth, M., Shogren, J. F. The benefits and costs of using social distancing to flatten the curve for COVID-19. Journal of Benefit-Cost Analysis. 2020; 11(2): 179–195. doi: https://doi.org/10.1017/bca.2020.12

9. **The section: Vaccine Cost Benefit Analysis not relevant to the purpose of this article...**

Answer As advised, this part has been deleted and we rewrote it as a research limitation in the discussion section.

→ "An economic cost and benefit analysis of mitigating policies such as social distancing and vaccination can be conducted using the results of various simulations in this study. Specifically, we can examine whether the economic benefits of social distancing (or vaccination) exceed or fall short of its costs. Since the Korean government has changed the intensity of social distancing several times in the course of fighting the spread of COVID-19, it can be a natural experiment to estimate the benefits and costs of the various intensity of social distancing.." (Lines 624-630)

10. **Chapter "Discussion" is poorly motivated in terms of biological interpretation. it does not provide any insight into how policymakers might use the model and the results.**

Answer We modified the Discussion section. We summarized the results obtained in the "Results" section and the facts that can lead to these results. The following is part of the Discussion section.

- We found in this experiment that non-pharmaceutical interventions lowered the number of confirmed cases and slowed the spread of transmission. A case where R_0 was actually reduced by the implemented policy was introduced in [50] and strategies for non-pharmaceutical interventions was introduced in [8]. (Lines 565-568)
- Even with vaccination, non-pharmaceutical interventions are still important. Figures 4, 6 show that with increasing R_0, we observe convergence to the unvaccinated state even with vaccination. As a detailed study related to this, there is a study [9]. (Lines 586-588)
- An interesting result of this experiment was that a new peak was produced when the effective reproduction number of the mutant virus was more than double that of the original virus. This could help us to allocate medical and administrative resources to two different viruses in the presence of mutant viruses. (Lines 591-594)
- As a result, we showed that a 10% change in vaccination rates can make a huge difference, reducing the number of confirmed cases by 35.22% or increasing it by 82.82%. These results may help inform people who are hesitant to get vaccinated. (Lines 609-612)

11. **What is about the limitations in the Discussion?**

Answer We added the limitation paragraph of the study to the Discussion section. A major limitation of our study is that reinfection was not taken into account.

→ We acknowledge some limitations of the present study. Our model ruled out reinfection between the two strain because the rate of reinfection was low at the time of the Delta variant in Korea [53]. We conducted simulations for the Omicron variant, which was newly reported toward the end of November 2021, by changing the parameters of the model and confirmed that the Omicron variant has a very large effective reproduction number, as shown in Figure 15. However, reinfection rates of the Omicron variant have been reported to be high [53] and our model was not suitable to provide a fitting and prediction for this variant in the long term. Also, we did not consider antibodies that disappear over time. This is very important for mutant viruses with high reinfection rates that may emerge in the future. We leave the model improvement considering reinfections for future research. (Lines 613-623)

12. **There is no analysis of fitting errors.**

Answer We added a relative L^2 norm error to each fitting section.

→ Lines 444, 484, and 540

Minor: the statements in the introduction require confirmation from the literature:

1. **the first statement - reference?**

Answer Severe acute respiratory syndrome coronavirus 2 (SARS-CoV-2), also known as COVID-19, emerged in late 2019 [1] and continues to threaten the world [2].

[1] Riou, J., Althaus, C. L. Pattern of early human-to-human transmission of Wuhan 2019 novel coronavirus (2019-nCoV), December 2019 to January 2020. Eurosurveillance. 2020;25(4): 2000058. doi: https://doi.org/10.2807/1560-7917.ES.2020.25.4.2000058

[2] Dong, E., Du, H., Gardner, L. An interactive web-based dashboard to track COVID-19 in real time. The Lancet infectious diseases 2020; 20(5), 533–534. doi: https://doi.org/10.1016/S1473-3099(20)30120-1

2. **second statement - reference?**

Answer The virus has reduced social activities such as traveling and interactions between individuals [3] and negatively impacted education [4] and the economy [5].

[3] UNWTO World Tourism Barometer and Statistical Annex, December 2020. UNWTO World Tourism Barometer (English version). 2020;18(7): 1–36. doi: 10.18111/wtobarometereng.2020.18.1.7

[4] UNESCO. Education: from school closure to recovery. Available: https://www.unesco.org/en/covid-19/education-response?hub=800

[5] World Bank. World Development Report 2022, Chapter 1. The economic impacts of the COVID-19 crisis. Available: https://www.worldbank.org/en/publication/wdr2022/brief/chapter-1-introduction-the-economic-impacts-of-the-covid-19-crisis

3. **Introduction: "Using the model above, we provide various simulations" - what model ?**

Answer We deleted that sentence.

4. **The last paragraph in Introduction is not clear...**

Answer We deleted that sentence in revised version of manuscript.

5. **"There are various methods of mathematical modeling of infectious diseases." - reference?**

Answer We changed the sentence to the following and added a citation.

→Diverse mathematical models exist for infectious diseases [32].

[32] Diekmann, O., Heesterbeek, J. A. P. Mathematical epidemiology of infectious diseases: model building, analysis and interpretation (Vol. 5). John Wiley & Sons. Chichester; 2000

6. **"The coupe model is one of the most representative methods of mathematical modeling" - reference?**

Answer We changed the sentence to the following and added a citation.

→ The compartment model is one of the representative mathematical modeling techniques [11].

[11] Banholzer, N., Van Weenen, E., Lison, A., Cenedese, A., Seeliger, A., Kratzwald, B., et al. Estimating the effects of non-pharmaceutical interventions on the number of new infections with COVID-19 during the first epidemic wave. PLoS one. 2021;16(6): e0252827. doi: https://doi.org/10.1371/journal.pone.0252827

7. **"To build our model, we will start with basic epidemic models, which are the prototypical epidemic models." – not clear?**

Answer We deleted that sentence in revised version of manuscript.

8. **"As an epidemic occurs, the number of susceptible people decreases over time and eventually becomes zero." – not agree. . .**

Answer We deleted that sentence in revised version of manuscript and we cited the study [24] in the introduction.

→ [24] Lazebnik, T., Bunimovich-Mendrazitsky, S., Shaikhet, L. Novel method to analytically obtain the asymptotic stable equilibria states of extended SIR-type epidemiological models. Symmetry. 2021;13(7): 1120. doi: https://doi.org/10.3390/sym13071120

4 Reviewer 4

A model of COVID-19 pandemic with vaccines and mutant viruses The author has worked on a model of the COVID-19 pandemic with the pres- ence of multiple mutant viruses and studied the effect of vaccination on diease propagation in South Korea. They have developed a mathematical model based on the SEIR type of compartment model in which they have added the various compartments related to multiple vaccinations and mutant virus of COVID-19. The author has calculated the effective reproduction numbers and equilibrium points of the original virus, Delta, and Omricon strains of the SARS-CoV-2 virus. Further, the author has carried out numerical simulations for the differ- ent cases and fit the model with the observed data from the confirmed cases from South Korea. The author has did a good work. I will suggest the following modifications in the manuscript.

1. **I will suggest the author to rewrite the introduction . In the introduc- tion , the author should cite some more literatures related to modeling of COVID-19 disease transmission with the presence of multiple mutant viruses and the effect of vaccination. Also, the author should include some works related to multistrain dynamics of various strains of SARS-CoV-2 virus.**

Answer As advised, we have rewritten the Introduction section. We reviewed the latest research related to COVID-19 modeling, multi-strain models, and papers related to vaccination.

2. **In the method part, it is not necessary to explain about SIR and SEIR model. The author can directly start explaining the proposed SEIHRV model which is based on SEIR framework. They can cite the original reference of SEIR model while writing the method.**

Answer We agree with what you pointed out. So, we deleted the parts related to SIR and SEIR model from the Method section and cited the related papers in the Introduction section.

3. **The author should explain little bit about the breakthrough infection in the model.**

Answer We confused the terms reinfection and breakthrough infection and used them incorrectly. Therefore, the assumption part of the model (Line 97) was modified. The added sentence is as follows:

When a vaccine does not provide complete immunity to the virus and the vaccinated individual becomes infected with the disease, it is called "breakthrough infection."

The above sentence is quoted from [CDC (2020-02-11). "COVID-19 Vaccination". Centers for Disease Control and Prevention. Retrieved 2022-01-06].

4. **In Figure 2., the author should slightly modify the schematic diagram for SEIHRV model. There is an extra I m compartment near the vaccine compartments (V_1, V_2 and V_3).**

Answer We drew Figure2 again to avoid confusion.

5. **As the COVID-19 pandemic is endemic now in the world, I think the author should include the demographic information in model such as birth rate and death rate.**

Answer As you pointed out, we modified the model to reflect births and natural deaths. Λ added to the model represents the number of births and ν represents the natural death rate. These are summarized in Table2 (Line 163). We have modified our assumptions because of this.

The changed sentence is as follows :

Because the COVID-19 pandemic has manifested for a long period, our model considers the natural mortality and birth rates of the population in the course of the pandemic. (Line 93)

6. **In Figure. 11, the author should mentioned the values of τ each of the sub-figures from 1.1 to 1.8.**

Answer We added each appropriate tau to each sub-figure. Also, since the starting point of tau is different from the manuscript, we changed it to 1.3.
