# Supplementary material for: A model of COVID-19 pandemic with vaccines and mutant viruses
Source: PLoS One. 2022 Oct 24;17(10):e0275851. doi: 10.1371/journal.pone.0275851 (PMC9591069; doi:10.1371/journal.pone.0275851)
Supplement: Attachment — Submitted filename: extended SIR with mutation - review.docx [file pone.0275851.s004.docx]

The authors proposed an extended SIR with mutation and a multi-vaccine model.

In general, the idea is good and the implementation is on top. I like their work.

This paper has some publishing points, but there are several issues why this paper is not ready for publication.

Main:

1. Extensive editing of English language and style required. The text is difficult to read due to grammatical and linguistic problems found in the article.
2. The abstract does not report any findings, only an approach. What is the novelty of the manuscript?
3. The biological introduction and the review of existing modelling is insufficient.

The authors should discuss recent work on multi-strain, multi-mutation models and other models combining SIR and vaccination.

There are several recent publications in PLOS one:

***D. Arruda et al., Modeling and optimal control of multi-strain epidemics with application to COVID-19 (2021) - Plos. one.***

***A. Marchioni and de Aguiar, Modeling Neutral Viral Mutations in the Spread of SARS-CoV-2 Epidemics (2021) - Plos one.***

***B. Lazebnik and Bunimovich-Mendrazitsky, A general approach to the mathematical model of multi-strain pandemics, (2022) - Plos one.***

***C. Gordo et al., Genetic Diversity in the {SIR} Model of Pathogen Evolution (2009) - Plos One.***

4. "It allocates the population to labeled compartments, such as S, I, or R, 81 (susceptible, infectious, or recovered)." - bad wording and quote! it's not your idea...

5. The equations 1+2 are known, not need speak about it…

6. Explanation of the equations (3) must be provided!.

8. N = 50, 000, 000, σ = 1/4.1 and µ = ¼ - you must explain these parameters!

9. No clear explanation of the graphs. Authors should provide analysis of these results.

10. The section: Vaccine Cost Benefit Analysis not relevant to the purpose of this article...

11. Chapter "Discussion" is poorly motivated in terms of biological interpretation. it does not provide any insight into how policymakers might use the model and the results.

12. What is about the limitations in the Discussion?

13. There is no analysis of fitting errors.

Minor: the statements in the introduction require confirmation from the literature:

1.the first statement - reference?

2. second statement - reference?

3. Introduction: "Using the model above, we provide various simulations" - what model ?

4. The last paragraph in Introduction is not clear...

5. "There are various methods of mathematical modeling of infectious diseases." - reference?

6. "The coupe model is one of the most representative methods of mathematical modeling" - reference?

7. “To build our model, we will start with basic epidemic models, which are the prototypical epidemic models.” – not clear?

8. “As an epidemic occurs, the number of susceptible people decreases over time and eventually becomes zero.” – not agree…
